# Molecular Basis of Complement C1q Collagen-Like Region Interaction with the Immunoglobulin-Like Receptor LAIR-1

**DOI:** 10.3390/ijms22105125

**Published:** 2021-05-12

**Authors:** Guillaume Fouët, Isabelle Bally, Anne Chouquet, Jean-Baptiste Reiser, Nicole M. Thielens, Christine Gaboriaud, Véronique Rossi

**Affiliations:** Institute of Structural Biology (IBS), Univ. Grenoble Alpes, CEA, CNRS, IBS, F-38000 Grenoble, France; gui.fouet@gmail.com (G.F.); isabelle.bally@ibs.fr (I.B.); anne.chouquet@ibs.fr (A.C.); jean-baptiste.reiser@ibs.fr (J.-B.R.); nicole.thielens@ibs.fr (N.M.T.)

**Keywords:** immune tolerance, immunoglobin-like receptor, complement C1q, collagen-like region

## Abstract

The immune system homeostasis relies on a tight equilibrium of interconnected stimulatory and inhibitory signals. Disruption of this balance is characteristic of autoimmune diseases such as systemic lupus erythematosus (SLE). Aside from activating the classical complement pathway and enhancing pathogens and apoptotic cells phagocytosis, C1q has been recently shown to play an important role in immune modulation and tolerance by interacting with several inhibitory and stimulatory immune receptors. Due to its functional organization into collagen-like (CLR) and globular (GR) regions and its multimeric nature, C1q is able to interact simultaneously with several of these receptors and locally congregate pro- and anti-inflammatory signals, thus modulating the immune response. Leukocyte associated immunoglobulin-like (Ig-like) receptor 1 (LAIR-1), a ubiquitous collagen receptor expressed in many immune cell types, has been reported to interact with the CLR of C1q. In this study, we provide new insights into the molecular and structural determinants underlying C1q/LAIR-1 interaction. Recombinant LAIR-1 extracellular Ig-like domain was produced and tested for its interaction with C1q. A molecular dissection of C1q combined with competition assays reveals that LAIR-1 interacts with C1q’s CLR through a binding site close but different from the one of its associated C1r2s2 proteases tetramer. On the other side, we identified LAIR-1 residues involved in C1q interaction by site-directed mutational analysis. All together, these results lead to propose a possible model for C1q interaction with LAIR-1 and will contribute to the fundamental understanding of C1q-mediated immune tolerance.

## 1. Introduction

The ability of the immune system to provide an efficient response against pathogens without hyper reacting against host tissues relies on a tight balance between pro- and anti-inflammatory signals. Disruption of this equilibrium can lead to autoimmunity. C1q, a protein initially identified as the recognition protein that triggers the classical pathway of the complement system, has been shown to play a major role in the maintenance of immune tolerance [1]. Indeed, homozygous deficiencies in C1q are strongly associated with severe autoimmune inflammatory diseases such as systemic lupus erythematosus (SLE) (>90% prevalence) [2]. C1q is a multimeric protein composed of 18 polypeptide chains of types A, B and C. Assembled in six heterotrimers maintained by both noncovalent and covalent interactions, these chains are composed of a short N-terminal part containing interchain disulfide bonds (between A-B and C-C), followed by a collagen-like region (CLR) containing the characteristic repeating G-X-Y triplets (X being any amino acid and Y a proline or hydroxyproline residue) and a C-terminal globular region (GR) [3]. This complex assembly leads to the formation of a 460 kDa C1q protein with a “bouquet-like” structure, where six C-terminal globular domains are connected via six collagen stems gathered in an N-terminal fiber bundle [4,5]. Functional studies revealed distinct activities for the GR and CLR of C1q. On one hand, the globular regions are mostly involved in the recognition of several targets like antibodies Fc region, molecules on pathogens surfaces or altered-self components [6,7]. On the other hand, the collagen-like regions of C1q are engaged in immune response effector mechanisms through their interaction with a tetramer of complement C1r and C1s proteases (C1r_2_s_2_) or receptors on immune cells surface [8,9].

Leukocyte associated immunoglobulin-like receptor 1 (LAIR-1; CD305) is an inhibitory receptor of 32 kDa expressed on many types of immune cells and composed of an extracellular C2-type immunoglobulin-like (Ig-like) domain, a single transmembrane segment and a cytoplasmic tail containing two immunoreceptor tyrosine inhibitory motifs (ITIMs) [10,11]. It is well established that LAIR-1 is a receptor for a large range of collagen types, from extra cellular matrix and transmembrane collagens (such as collagens I, II, III, IV, XIII, XVII or XXIII) to collagen-containing proteins such as surfactant protein D (SP-D), mannose-binding lectin (MBL) or C1q. LAIR-1 activation by one of these identified ligands leads to its ITIMs phosphorylation, recruitment of SHP-1 tyrosine phosphatase and inhibition of proinflammatory signals [12,13,14,15,16]. Human LAIR-1 has a soluble homologue LAIR-2 shown to interact with the same ligands and supposed to control LAIR-1 inhibitory activity by competing for collagen binding [12,13,17]. LAIR-1 is the only known inhibitory receptor of C1q CLR and Son and colleagues have reported that it is an important mediator of C1q-mediated immune tolerance, together with other immune receptors such as CD33 and the receptor for advanced-glycation end-products (RAGE) [14,18,19]. Due to its multimeric nature, C1q would likely interact with several of these receptors simultaneously, thus generating local gathering of pro- and anti-inflammatory signals. However, these processes remain unclear and the molecular and structural determinants of C1q interaction with these receptors have not yet been identified.

In the present study, we aimed at deciphering the molecular basis of C1q/LAIR-1 interaction. To do so, we produced recombinant soluble LAIR-1 Ig-like domain and tested its interaction properties for C1q by surface plasmon resonance (SPR). We showed that C1q interacts with LAIR-1 via its CLR on a binding site close to, but different from the one of C1r_2_s_2_. On LAIR1, mutational analyses allowed the identification of several key residues involved in C1q interaction. Taken together, these results lead us to propose a model for the C1q/LAIR-1 interaction.

## 2. Results

### 2.1. Recombinant LAIR-1 Ig-Like Domain Interacts with Serum-Derived C1q

The recombinant soluble Ig-like domain of wild-type LAIR-1 (WT) and all the variants used in the present study were expressed in bacteria and purified as described in Section 4. LAIR-1 extracellular domain contains one N-glycosylation site at the asparagine residue 69. For that reason, LAIR-1 extracellular region was also produced in HEK293F cells to assess its binding and the importance of the LAIR-1 glycosylation. LAIR-1 Ig-like domain produced in E. coli or HEK293F cells showed similar levels of interaction with C1q (Figure S1) but with slight differences in the binding curves possibly accounting for posttranslational modifications. Consequently, the results presented in this study were obtained using LAIR-1 produced in bacteria.

**Figure 1:**
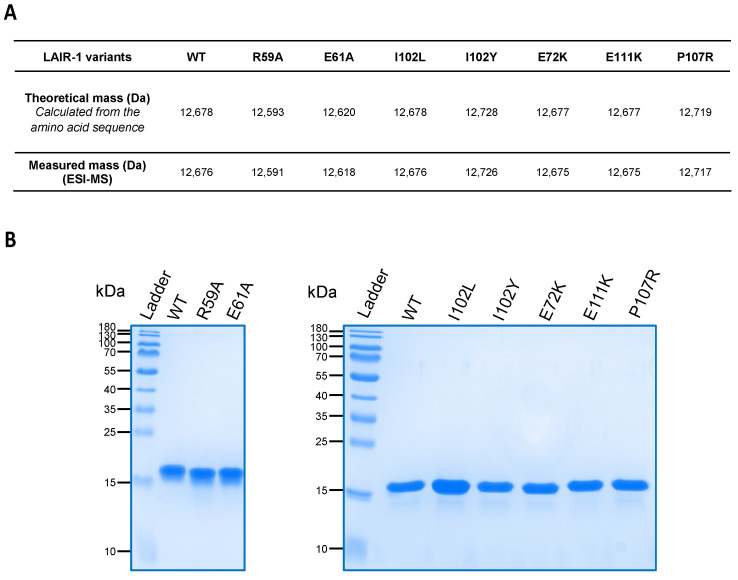
Quality control of the LAIR-1 Ig-like domain variants. (**A**) Summary table of the theoretical masses calculated from the amino acid sequences and the measured masses by ESI-MS experiments of the LAIR-1 variants produced. (**B**) SDS-PAGE analysis of the LAIR-1 variants on a 14% polyacrylamide gel under reducing condition.

The quality of each LAIR-1 variant was analyzed by ESI-MS and SDS-PAGE (Figure 1). As illustrated in Figure 1A, each LAIR-1 Ig-like domain variant exhibited an ESI-measured mass in agreement with the theoretical mass calculated from the amino acid sequence. Of note, a difference of 2 Da is observed between the theoretical and measured masses accounting for the presence of an intact disulfide bond, thus supporting the correct folding of the Ig-like domain. As expected, SDS-PAGE analysis of LAIR-1 WT and mutated samples reveals a unique protein band with an apparent molecular weight of about 15 kDa, consistent with masses determined by mass spectrometry (Figure 1B).

**Figure 2:**
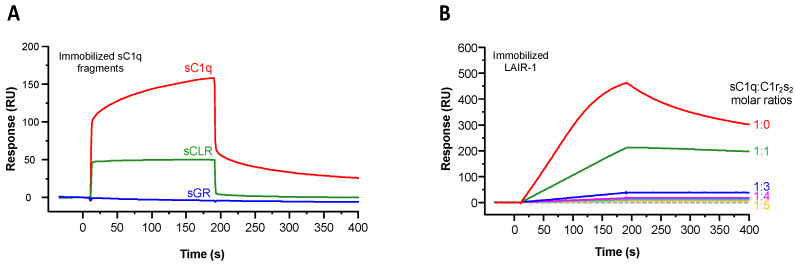
Identification of the C1q functional region interacting with LAIR-1 and competition assay with the C1r_2_s_2_ tetramer. (**A**) LAIR-1 (10 µM) was injected over immobilized serum-derived C1q (sC1q, red curve), sCLR (green curve) or sGR (blue curve). (**B**) sC1q (1 nM) was preincubated with C1r_2_s_2_ at increasing molar ratios (1–5) and injected over immobilized LAIR-1. The grey dashed line represents the C1r_2_s_2_ tetramer injection at a concentration of 10 nM as a control.

We first assessed the interaction of the recombinant LAIR-1 Ig-like domain with C1q by surface plasmon resonance (SPR). LAIR-1 interacts with serum-derived C1q (sC1q) in two SPR configurations, i.e., soluble LAIR-1 on immobilized sC1q or soluble sC1q on immobilized LAIR-1 (red curves in Figure 2A,B, respectively). However, the difference in the curve shapes of the two configurations suggests avidity effects in the interaction of C1q with immobilized LAIR-1, which is absent in the other configuration, when C1q is immobilized. This likely reflects the presence of multiple LAIR-1 binding sites on the sC1q molecule. In order to avoid avidity constraints in SPR analyses, we decided to perform all SPR experiments using immobilized C1q fragments, with the exception of the C1r_2_s_2_ competition assay for which the injection of soluble sC1q is required.

### 2.2. LAIR-1 Interacts with C1q Collagen Stems Through a Site in Close Proximity but Different from the C1r2s2 Tetramer Binding Site

In order to identify which C1q functional region is involved in LAIR-1 interaction, soluble LAIR-1 Ig-like domain was injected over immobilized serum-derived GR or CLR. As illustrated in Figure 2A, LAIR-1 interacts with both sC1q and sCLR but no interaction signal was detected on immobilized sGR. This result reveals that LAIR-1 Ig-like domain interacts with C1q exclusively through its collagen-like regions.

With a view to locate more precisely the LAIR-1 binding site in the C1q collagen-like regions, a competition experiment was then performed with a C1q ligand known to interact with the CLR within the C1 complex: the C1r_2_s_2_ tetramer. For that purpose, the C1 complex was reconstituted by incubation of sC1q with recombinant C1r_2_s_2_ at increasing molar ratios before injection over immobilized LAIR-1. C1q was no more able to interact with LAIR-1 when it is fully associated with C1r_2_s_2_ (above 1:3 tetramer versus C1q molar ratio), revealing that the LAIR-1 binding site on C1q CLR might be the same or located in close proximity of the C1r_2_s_2_ binding site. Of note, the need for a molar ratio higher than 1:1 to fully inhibit C1q interaction with LAIR-1 can be explained by the fact that the concentration used (1 nM) is almost 10 times lower than the previously determined K_D_ of C1q interaction with C1r_2_s_2_ (around 13 nM; Bally et al. 2013) [20]. Importantly, such a total inhibition of LAIR-1 interaction with C1q when associated with C1r_2_s_2_ suggests that no other LAIR-1 binding site, distant from the C1r_2_s_2_ binding region, is present on C1q CLR (Figure 2B).

**Figure 3:**
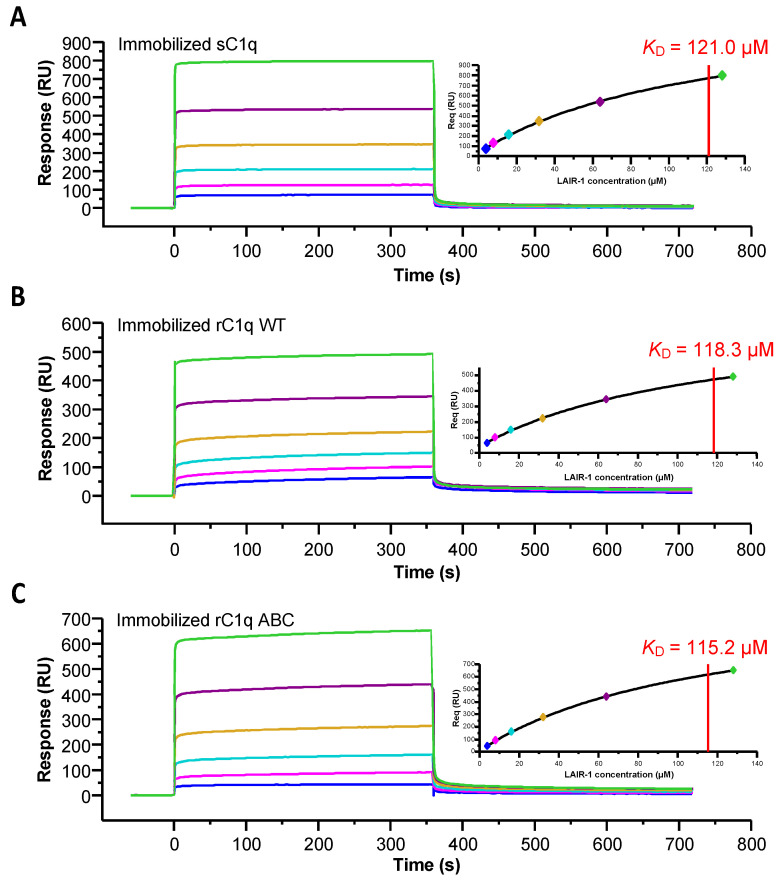
Kinetic analysis of LAIR-1 interaction with immobilized serum-derived C1q, rC1q WT and rC1q ABC. LAIR-1 was injected at increasing concentrations (4–128 µM, 2-fold serial dilution) on immobilized sC1q (**A**), rC1q WT (**B**) and rC1q ABC (**C**). Fits obtained by a global fitting of the data to a steady-state model are shown in the top right corner of each panel. The results shown are representative of three (sC1q) or two (rC1q WT and ABC) experiments (see Table 1).

As mentioned above, the C1r_2_s_2_ tetramer is known to interact with the collagen-like regions of C1q and C1q lysine residues are involved in this binding [20]. To assess if LAIR-1 binding to C1q CLRs also requires these lysine residues, we used a recombinant C1q mutant with an alanine substitution of LysA59, LysB61 and LysC58 (rC1q ABC) with recombinant wild-type C1q (rC1q WT) as a control. LAIR-1 binding to these two rC1q variants was assessed using SPR. Although rC1q ABC does not interact with C1r_2_s_2_, this variant retains its interaction properties with LAIR-1 with a K_D_ similar to that of rC1q WT and sC1q (Figure 3 and Table 1). This reveals that C1r_2_s_2_ and LAIR-1 interact with C1q through different binding sites.

**Figure 4:**
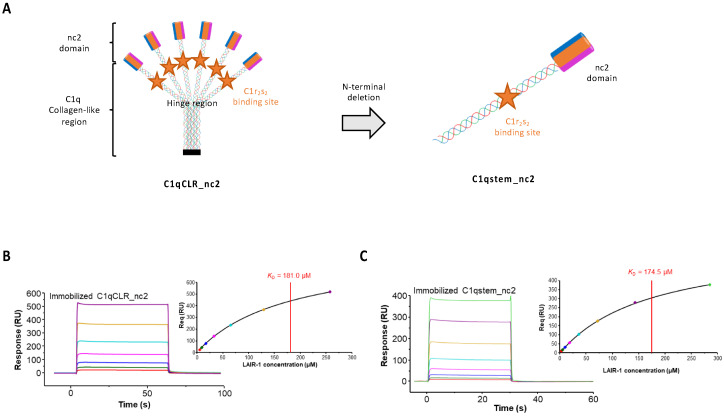
Analysis of LAIR-1 interaction with C1qCLR_nc2 and C1qstem_nc2. (**A**) Schematic representation of the full length C1qCLR_nc2 protein (left part) and C1qstem_nc2 fragment (right part), the C1r_2_s_2_ binding site is represented with an orange star. (**B**,**C**) Soluble LAIR-1 Ig-like domain was injected at increasing concentrations over immobilized C1qCLR_nc2 (**B**) and C1qstem_nc2 (**C**). Fits obtained by a global fitting of the data to a steady-state model are shown in the top right corner of each panel. The results shown are representative of three experiments.

As LAIR-1 interaction with C1q involves its collagen-like regions, we used a new C1qCLR_nc2 construct derived from the CLR_nc2 fusion protein recently developed in our laboratory to investigate the collagen-like functions of C1q [21]. We needed to delete the GPP triplets initially added between the CLR and nc2 sequences in order to avoid addition of artifactual LAIR-1 binding sites into the molecule (see Section 3). The ability of LAIR-1 Ig-like domain to interact with wild-type C1qCLR_nc2 was assessed by SPR. As expected, LAIR-1 WT interaction with C1qCLR_nc2 exhibited a K_D_ value in the same range as with sC1q (Figure 4B and Table 1), therefore confirming that C1q interacts with LAIR-1 via its CLR.

We went further in engineering the C1qstem_nc2 fusion fragment, obtained by deleting the N-terminal part of C1qCLR_nc2 until the CLR hinge region. This C1qstem_nc2 truncated fragment contains a single heterotrimeric C1q collagen stem comprising the C1r_2_s_2_ and LAIR-1 binding sites with one C-terminal nc2 domain (Figure 4A). The C1qstem_nc2 construction was tested for its interaction with LAIR-1 Ig-like domain using SPR and the results indicated that it interacts in a dose-dependent manner (Figure 4C). Kinetic analysis of this interaction reveals a K_D_ value similar to the one obtained with the full length C1qCLR_nc2. This confirms that the LAIR-1 binding site might not be located in the N-terminal collagen bundle.

All together, these results highlight that LAIR-1 interacts with C1q stems through a binding site close but different from the binding site for the C1r_2_s_2_ tetramer.

### 2.3. A Central Binding Groove in LAIR-1 Common for C1q and Generic Collagen Recognition

**Figure 5:**
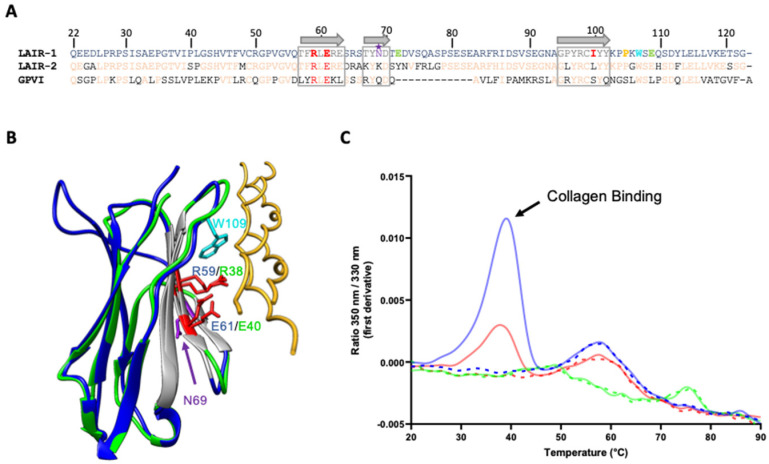
LAIR-1 R59 and E61 are central collagen binding residues, conserved in LAIR-2 and GPVI. (**A**) Sequence alignment of LAIR-1, LAIR-2 and GPVI Ig-like domains. The β-strands forming the collagen interaction groove in LAIR-1 are indicated with grey arrows and the N-glycosylation site (N69) is indicated with a purple star. The LAIR-1 mutated residues are colored in red for the central collagen binding site and in green and orange for the identified distal residues. LAIR-1 conserved and different residues in LAIR-2 and GPVI sequences are colored in light orange and black, respectively. (**B**) Alignment of the crystal structure of the Ig-like domains of LAIR-1 (in blue, PDB code: 3KGR) and GPVI (in green) in complex with a collagen peptide (in yellow) (PDB code: 5OU8). LAIR-1 R59 and E61 and the GPVI homologous residues are colored in red. LAIR-1 N69 is labeled in purple. The remarkable W109 in LAIR-1, close to the collagen ligand, is labeled in cyan. (**C**) NanoDSF experiments of LAIR-1 WT (in blue), R59A (in green) and E61A (in red), alone (dashed lines) or in the presence of a positive collagen peptide control (solid lines).

LAIR-1 is known to be a collagen receptor and previous studies identified a three-stranded β-sheet as the surface involved in the interaction of LAIR-1 Ig-like domain with collagens (in grey in Figure 5A,B). Among the residues forming this interaction groove, two LAIR-1 central amino acids, arginine 59 (R59) and glutamate 61 (E61) are essential for its binding to collagens I, III and IV [22]. Interestingly, these two important residues for collagen interaction are conserved in the homologous glycoprotein VI receptor (GPVI) involved in collagen-induced activation and aggregation of platelets (Figure 5A,B; [23]. Based on the assumption that LAIR-1 interaction with C1q CLR could be similar to the one with collagens I, III and IV, we produced LAIR-1 Ig-like domain variants with an alanine residue at positions 59 and 61 (R59A and E61A, respectively).

In order to investigate the mutation effects on the interaction of LAIR-1 with a generic collagen used as a positive control, we tested LAIR-1 interaction with a synthetic homotrimeric collagen peptide containing a central stabilizing GPKGEQ motif, found in several defense collagen sequences, surrounded by GPO triplets [24,25]. To do so, we took advantage of the fact that the collagen binding groove in LAIR-1 contains a unique exposed tryptophan residue (W109) (Figure 5B). Indeed, the interaction of the collagen peptide with LAIR-1 may thus change the tryptophan environment and induce a transition in its fluorescence properties that can be detected using nano-differential scanning fluorimetry (nanoDSF). As illustrated in Figure 5C, collagen peptide binding to LAIR-1 was assessed using nanoDSF measurements. This showed that both independent substitutions of R59 or E61 strongly impact LAIR-1 binding to the collagen peptide, with a higher effect for the R59A mutation.

**Figure 6:**
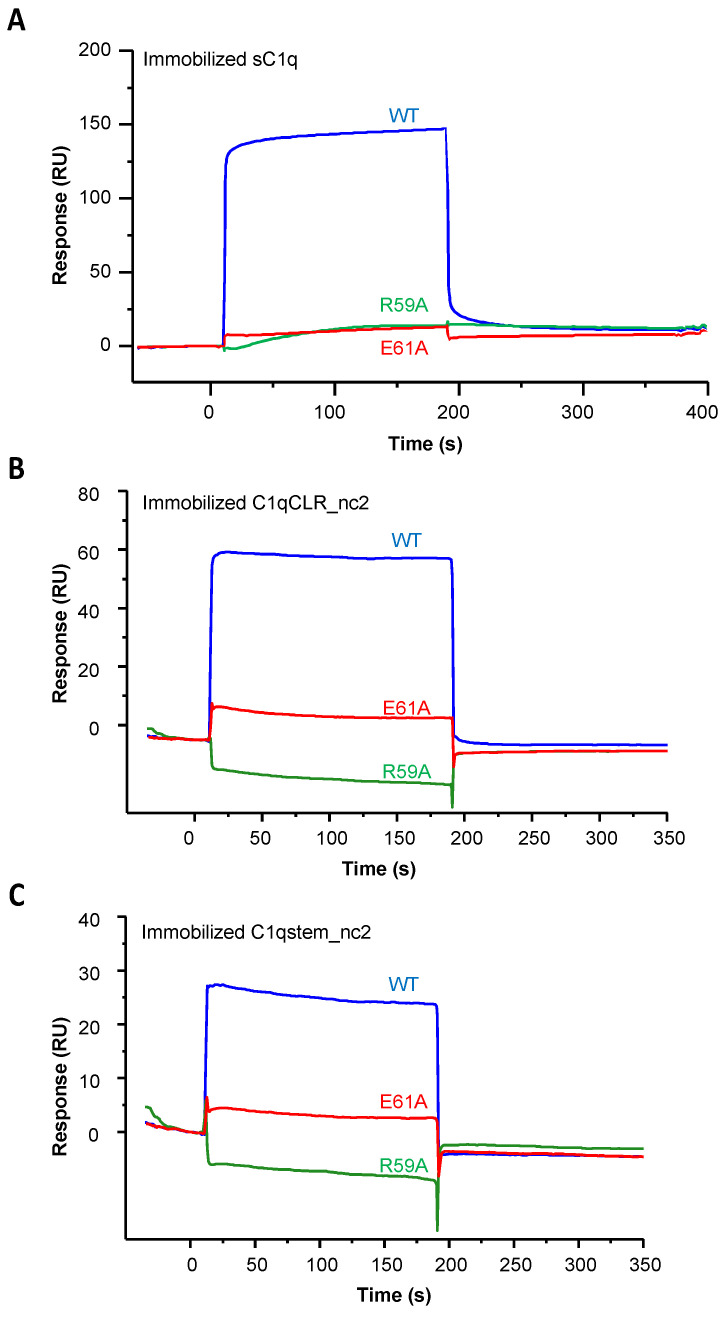
LAIR-1 R59A and E61A mutations abrogate the interaction with C1q and its variants. LAIR-1 WT (blue curves), R59A (green curves) and E61A (red curves) were injected at a concentration of 10 µM over immobilized sC1q (**A**), C1qCLR_nc2 (**B**) and C1qstem_nc2 (**C**).

**Figure 7:**
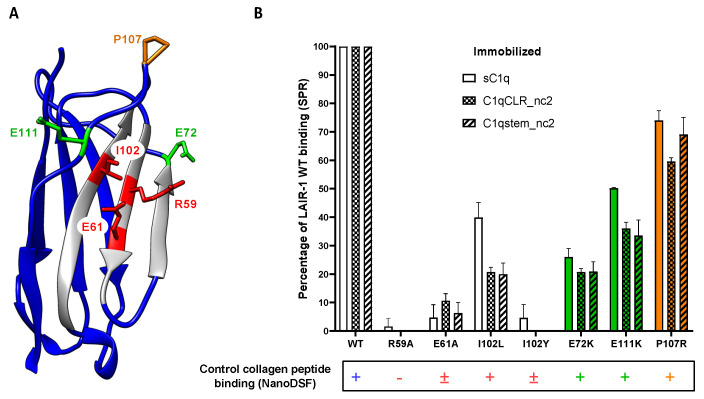
Mutational analysis of LAIR-1 interaction with C1q. (**A**) Crystal structure of LAIR-1 Ig-like domain (PDB code: 3KGR). The side chains of the mutated residues are shown and labeled. The central binding residues are colored in red and the distal residues are shown in green and orange (as in Figure 5). (**B**) SPR measurements of the interaction of LAIR-1 WT and mutants with sC1q, C1qCLR_nc2 and C1qstem_nc2. Results are expressed as the percentage of the LAIR-1 WT response and error bars represent the SD of three different experiments. The detection of a binding signal of LAIR-1 variants with the positive collagen peptide control measured with the nanoDSF method is reported at the bottom. The presence or absence of the characteristic binding signal is reported with + or − symbols, respectively. The cases where a weak binding signal remains are indicated with a ± symbol. The raw data of the peptide interactions are presented in Figure 5B and Figure S5.

SPR experiments on immobilized sC1q, C1qCLR_nc2 and C1qstem_nc2 showed that these substitutions of arginine 59 or glutamate 61 into an alanine residue almost completely abolish LAIR-1 interaction with C1q, pointing out that, as for the positive collagen peptide control, these two residues are essential for C1q interaction (Figure 6 and Figure 7B). The E61A mutation effect on LAIR-1 interaction with C1q fragments is slightly weaker than for the R59A mutation, which is in line with the nanoDSF experiments with a collagen peptide (Figure 5C, Figure 6 and Figure 7B). Circular dichroism (CD) experiments confirmed that these mutations do not affect folding, since both R59A and E61A variants displayed similar CD spectra to the WT LAIR-1 Ig-like domain (Figure S2).

These results unveil that C1q interaction with LAIR-1 is based on a generic collagen recognition by the two central arginine 59 and glutamate 61 LAIR-1 residues.

Previous reports showed that LAIR-1 and its soluble homologue LAIR-2 have different affinities for C1q [14,26] and comparison of LAIR-1 and LAIR-2 sequences highlights only few differences in the amino acids exposed on the β-sheet forming the interaction groove for collagen binding (Figure 5A). Among them, the isoleucine 102 of LAIR-1, which is in a central position in the interaction groove (Figure 7A), corresponds to a leucine residue in LAIR-2 sequence. Such substitution might induce a slight steric hindrance on the interaction surface, so we decided to introduce it in LAIR-1 (I102L) in order to investigate possible steric impact at that position. Isoleucine 102 was also replaced by a bulkier tyrosine residue (I102Y) in order to enhance the steric effect on collagen interaction. Interaction studies using SPR showed that, when isoleucine 102 is replaced by a slightly longer residue (I102L), the interaction with C1q is partially hampered and it is abolished in the case of the larger I102Y substitution (Figure 7B). However, the interaction with the control collagen peptide is only affected by the I102Y mutation (Figure 7B, lower part). This highlights that the isoleucine 102 position is prone to steric conflict for C1q binding, revealing that the collagen residue placed in front of the I102 of LAIR-1 should be small enough to accommodate LAIR-1 binding without introducing steric conflict.

### 2.4. Building of a C1q/LAIR-1 Interaction Model

**Figure 8:**
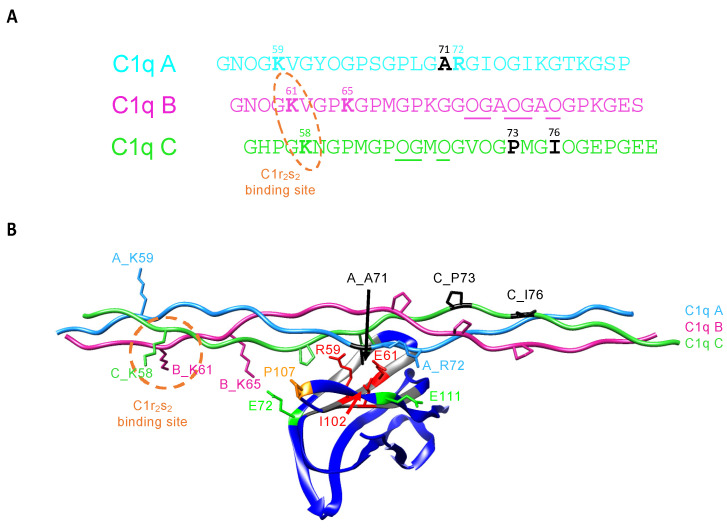
Model of LAIR-1 Ig-like domain in complex with a C1q collagen stem. (**A**) Sequences of C1q A, B and C chains corresponding to the C1q collagen-like region shown in B. The three lysine residues constituting the C1r_2_s_2_ binding site (A_K59, B_K61 and C_K58) are in bold and the two essential lysines are surrounded with a dashed orange ellipse. The putative LAIR-1 binding OGXO motifs in this region are underlined. The remarkable residues are labeled with their position numbers. The three residues facing the LAIR-1 I102 in each alternative model are labeled in black. The “O” in the sequences stands for proline residues modified into hydroxyprolines, as determined in the literature [27,28]. (**B**) Model of LAIR-1 Ig-like domain (blue, PDB code: 3KGR) in interaction with the C1q CLR triple helix (A chain in cyan, B chain in pink and C chain in green). The collagen-binding groove of LAIR-1 is shown in grey and the side chains of the identified key binding residues are shown and labeled. Mutated lysine 65 and arginine 72 of C1q B and A chains, respectively, are labeled. The residues facing LAIR-1 I102 in each model are colored in black. The side chains of the identified OGXO motifs are shown in lines.

As mentioned above, the central LAIR-1 arginine 59 and glutamate 61 residues are essential for C1q but also generic collagen backbone binding. Nevertheless, we showed that LAIR-1 recognition of C1q does not randomly occur along the CLR but is located on a specific binding site close to the C1r_2_s_2_ interaction region. Therefore, we investigated the binding specificity of C1q interaction with LAIR-1. For that purpose, we designed a model based on the structural literature and the results presented above. One crystal structure of GPVI in complex with a GPO-containing collagen peptide is available in the protein data bank (PDB code: 5OU8). Analysis of this structure reveals that the GPVI R38 and E40 residues (homologues of LAIR-1 R59 and E61, respectively) are central in collagen interaction and recognize the OGXO motif of two consecutive GPO triplets of a single chain. LAIR-1 R59 and E61 residues should likely recognize similar motifs on C1q. We identified three similar motifs in the C1q B and C chain sequences in close proximity to the C1r_2_s_2_ binding site that could be recognized by LAIR-1 (underlined in Figure 8A). We performed a structure alignment of the LAIR-1 Ig-like domain (PDB code 3RP1) with the GPVI collagen-binding domain and aligned the GPVI-associated collagen peptide with a model of one C1q collagen stem, in the region of the putative LAIR-1 binding sites on their common hydroxyproline residues. Such alignment led to the generation of three putative models of LAIR-1 in interaction with one C1q stem (Figure 8 and Figure S3). In the different models, we identified three residues facing the LAIR-1 I102 residue: A71 on C1q A chain, P73 and I76 on C1q C chain (represented in black in Figure 8 and Figure S3). Considering the constraint that a small C1q residue must face LAIR-1 I102, together with the close proximity to the C1r_2_s_2_ and the presence of two possible electrostatic interactions at side of the central R59 and E61, the model represented in Figure 8B with an alanine residue at this position on C1q A chain (A_A71) was selected as the most consistent one.

### 2.5. Exploring the Distal LAIR-1 Residues Involved in C1q Binding

Interestingly, in the selected model shown in Figure 8B, two glutamate residues at positions 72 and 111 of LAIR-1 (E72 and E111) are facing two positively charged C1q residues: lysine 65 of C1q B chain (B_K65) and arginine 72 of C1q A chain (A_R72), respectively. These four residues might likely lock the specific recognition of the central C1q hydroxyproline residues by LAIR-1 R59 and E61. Indeed, these two pairs of charge-opposed residues are located at each side of the collagen binding groove of LAIR-1 and could introduce more specificity toward C1q than generic collagen recognition (Figure 7A and Figure 8B). In order to test the engagement of the two LAIR-1 glutamate residues, we generated two LAIR-1 variants. The SPR results show that both mutations reduce LAIR-1 binding to C1q and CLR_nc2 constructions, with a stronger effect for E72K than for E111K mutation (Figure 7B). However, these two mutations on LAIR-1 have no effect on LAIR-1 interaction with the control collagen peptide (Figure 7B, lower part). This suggests that the substitution of these two LAIR-1 glutamate residues more specifically impact the recognition of C1q by LAIR-1, possibly through C1qA_R72 and B_K65 basic residues.

**Figure 9:**
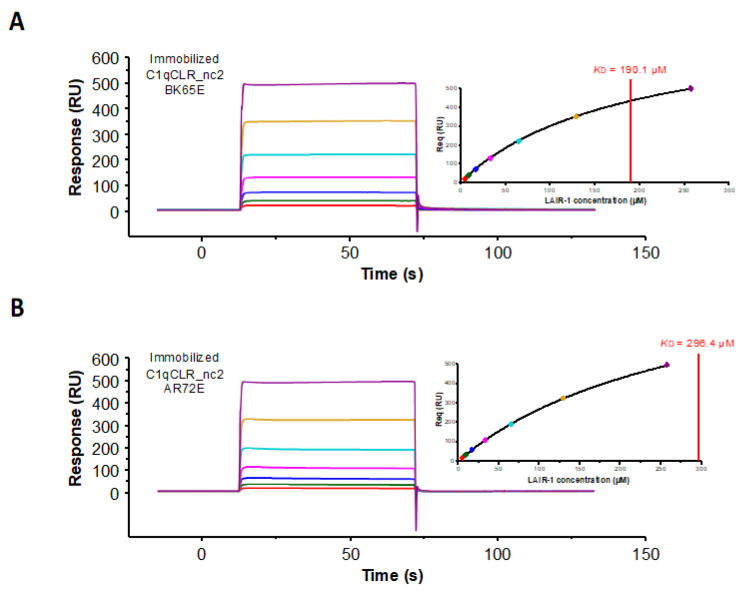
Kinetic analysis of LAIR-1 interaction with immobilized C1qCLR_nc2 variants. LAIR-1 was injected at increasing concentrations (4–256 µM, 2-fold serial dilution) over immobilized C1qCLR_nc2 B_K65E (**A**) and C1qCLR_nc2 A_R72E (**B**). Fits obtained by a global fitting of the data to a steady-state model are shown in the top right corner of each panel. The results shown are representative of three separate experiments (see Table 1).

In order to test the engagement of these two basic C1q residues in the interaction with LAIR-1, we generated two C1qCLR_nc2 variants containing the substitution of lysine 65 on C1q B chain and arginine 72 on C1q A chain into glutamate residues (B_K65E and A_R72E, respectively). SPR analyses showed that the B_K65E mutation did not have any effect on LAIR-1 interaction in comparison with the C1qCLR_nc2 WT (Figure 9A and Table 1). Regarding the C1qCLR_nc2 A_R72E variant, the K_D_ value obtained for LAIR-1 interaction was slightly higher than for C1qCLR_nc2 WT, suggesting that the arginine 72 on C1q A chain, albeit not essential, is likely involved in LAIR-1 interaction (Figure 9B and Table 1). These results can be related to the interaction behavior of LAIR-1 mutants of the two E72 and E111 residues. Indeed, the LAIR-1 E72K and E111K mutations exhibited weaker inhibitory effect on C1q interaction than the mutations in the central LAIR-1 binding site (i.e., R59 and E61; Figure 7). It is therefore consistent that the mutation of the suggested CLR residues facing the E72 and E111 residues of LAIR-1 triggers a weak effect on the global affinity of C1qCLR_nc2/LAIR-1 interaction, explained by their distal position from the central binding site of LAIR-1 (Figure 7B). We suggest that these two residues are not involved in the direct binding with LAIR-1 but lock and give the specificity of the interaction after initial LAIR-1 docking through its R59 and E61 residues.

Moreover, in order to go further in the deciphering of the LAIR-1 and C1q interaction, we studied the implication of residues that were described as important for the interaction with collagen in a family of broadly reactive antibodies against malaria antigens containing LAIR-1 Ig-like domain [29,30]. Indeed, these antibodies present a unique feature with the insertion of a full additional LAIR-1 Ig-like domain displaying several mutations that all together enhance the binding to *P. falciparum* infected erythrocytes while reducing the interaction with collagen. Interestingly, in almost all identified mutation profiles, arginine 59 and glutamate 61 constituting the LAIR-1 main collagen-binding site are conserved in the inserted LAIR-1 domain. One could wonder why these Ig-like domains do not interact with collagens, although they contain key residues for their recognition. Among all mutations, we paid attention to the substitution of proline 107 by an arginine residue (P107R). Indeed, this unique mutation leads to a LAIR-1 Ig-like domain devoid of its collagen binding properties [30]. We thus decided to produce this LAIR-1 Ig-like domain variant and assess its interaction either with C1q, C1qCLR_nc2 fragments or the control collagen peptide. As expected, this mutation reduced the interaction of LAIR-1 with C1q but had almost no effect with the positive control synthetic peptide (Figure 7B). This can be explained by the fact that the P107R mutation introduces a steric hindrance at the extremity of the collagen-binding groove of LAIR-1 Ig-like domain, which does not allow any longer the proper positioning of large collagen molecules like C1q, but retains the main central region for the binding to a smaller collagen peptide.

## 3. Discussion

Besides its major role in the initiation of the classical pathway of the complement cascade, C1q achieves many complement-independent functions such as the opsonization of apoptotic cells and pathogens or maintenance of immune tolerance. Therefore, C1q interaction network on immune cells surface is highly complex and the underlying molecular mechanisms need to be explored. In this study, we investigated the molecular basis of C1q interaction with LAIR-1, an inhibitory immune receptor present on various immune cells.

Molecular dissection of C1q unambiguously showed that LAIR-1 Ig-like domain interacts with the C1q collagen-like regions on a binding site located close to the one of C1r_2_s_2_ as shown by SPR competition assays. In addition, the interactions of LAIR-1 with recombinant C1q with a wild-type sequence or mutated at the C1r_2_s_2_ binding site were similar, revealing that LAIR-1 binding does not engage the same C1q CLR residues as C1r_2_s_2_. Interestingly, we showed that LAIR-1 interaction with C1q is only possible when it is devoid of its associated proteases. This result is in line with previous studies on C1q receptors showing that CR1 and LRP1 can interact with C1q but not with the C1 complex (i.e., C1q in complex with the C1r_2_s_2_ tetramer), showing that these three receptors share the same conditional rule for C1q binding and might share a common binding region on the C1q collagen stems [31,32].

This highlights the dual role of C1q in complement related function when associated with C1r_2_s_2_ and complement independent functions when devoid of its cognate proteases, through the interaction with cellular receptors. Even if serum C1q is almost exclusively found associated with C1r_2_s_2_ within the C1 complex, it is not unusual to find C1q alone in physiological conditions. Namely, single C1q can be locally expressed by several cell types or remain after C1-inhibitor mediated C1 complex dissociation [33,34]. C1q production by tissue-resident monocyte-derived cells is a process tightly controlled by numerous stimuli. For instance, C1q synthesis by macrophages was shown to be increased in response to tissue injury and constitutes a key process for resolution of inflammation [35].

Our study used C1qCLR_nc2, a variant of the CLR_nc2 recombinant fusion protein designed in our lab, previously reported to retain the C1r_2_s_2_ interaction properties of C1q CLR [21]. In the particular case of LAIR-1 study, we had to remove the three additional GPP collagen triplets initially introduced between the CLR and non-collagenous domain to improve the C1qCLR_nc2 folding. This GPP removal was done in order to avoid introducing artificial interaction sites for LAIR-1. It is likely that during processing and maturation, these additional GPP triplets undergo proline hydroxylation as post-translational modification, resulting in GPO collagen triplets. It is well established that GPO triplets are high-affinity ligands of LAIR-1 and such additional putative binding sites would have introduced nonspecific signal of LAIR-1 binding to CLR_nc2 [12,13]. Indeed, kinetics SPR experiments using the previously published CLR_nc2 protein containing the additional GPP triplets displayed a lower *K*_D_ value and a higher surface binding capacity (Rmax) substantiating the presence of additional binding sites and an increased avidity (Figure S4). Therefore, all experiments presented in this study were done using the C1qCLR_nc2 devoid of additional GPP triplets.

We report here that LAIR-1 interacts similarly with C1qCLR_nc2 and sC1q, thus validating the use of C1qCLR_nc2 in the context of studies on C1q collagen-like regions’ receptors. By deleting the N-terminal portion of each C1q collagen chain until the hinge region, we managed to produce one isolated heterotrimeric C1q collagen stem (called C1qstem_nc2). We showed that the affinity constants of LAIR-1 Ig-like domain interaction with C1qstem_nc2 and the LAIR-1 mutations effects are similar to those obtained with full length C1qCLR_nc2 (Figure 4B,C and Figure 7 and Table 1). This unambiguously excludes putative LAIR-1 binding sites in the C1q N-terminal collagen bundle even though recently reported results suggested the opposite. Indeed, Liu and colleagues showed that a molecular mimic combining short sequences of HMGB1 and the N-terminal region of C1q A chain was able to interact simultaneously with RAGE and LAIR-1 and efficiently polarize monocytes to an anti-inflammatory phenotype [36]. We suggest that this C1q A chain sequence, containing one PGXP motif, can be recognized by LAIR-1 in the context of the minimal structure of this mimic but is not accessible in the complex C1q architecture, probably hidden by the other C1q collagen stems forming the bundle. Furthermore, the generation of a short and functional heterotrimeric C1q collagen stem opens the way for various applications. For example, C1qstem_nc2 could be used for structural studies of C1q CLR ligands. This approach could be enlarged to other collagen containing proteins such as the soluble defense collagens family.

Moreover, a directed mutational analysis of LAIR-1 Ig-like domain based on the literature knowledge on LAIR-1 interaction with collagens, allowed a precise mapping of LAIR-1 residues involved in the interaction with C1q CLR. Indeed, we identified a C1q binding groove in LAIR-1 where the central residues (R59 and E61) directly interact with the collagen backbone and more distal residues (I102, E72, E111 and P107) are likely involved in the recognition specificity and the correct positioning of the C1q CLR in front of the LAIR-1 binding region. Our results are in agreement with a previous study identifying LAIR-1 key residues for collagen interaction [22]. The crystal structure of the GPVI in complex with a collagen peptide recently deposited in the PDB (code 5OU8) reveals that GPVI recognition of the collagen GPO motifs relies on two central arginine and glutamate residues homologous to the R59 and E61 of LAIR-1. Therefore, LAIR-1 and GPVI may share a common interaction mechanism with collagens, based on the direct recognition of the collagen through their central arginine and glutamate residues.

LAIR-2 is a soluble collagen receptor that interacts with the same ligands as LAIR-1 and is suggested to act as a competitor to regulate LAIR-1 collagen-induced inhibitory activity. However, the comparison of the affinity values of LAIR-1 and LAIR-2 interaction with C1q still deserves further investigation. Indeed, Son and colleagues previously reported that LAIR-1 exhibits more affinity for C1q than LAIR-2 while Olde Nordkamp et al. showed the opposite [14,26]. One contribution for the difference could be that the results of these two studies were obtained using LAIR proteins in different oligomeric states. Indeed, the first team used monomeric Ig-like domains whereas the latter used Fc-fused dimeric Ig-like domains. Since we observed that in the collagen binding groove I102 is substituted by a leucine in LAIR-2, we wanted to test the effect of this substitution on C1q binding in a LAIR-1 context, and generated the LAIR-1 I102L variant. We showed that C1q interaction with the LAIR-1 I102L variant is decreased, a result in line with the study of Son et al., also obtained using monomeric LAIR-1. It is noteworthy that within the GPVI, the corresponding residue of I102 is a serine, which is quite smaller. This suggests that the specific steric binding constraint for the residue facing I102 in LAIR-1 is likely missing for GPVI binding (Figure 5A), even if LAIR-1 and GPVI partly share a common collagen recognition mechanism.

The likely similar mechanism of collagen backbone recognition by LAIR-1 and GPVI allowed us to identify three putative LAIR-1 binding motifs on one C1q collagen stem (underlined in Figure 8A). We generated three different models of LAIR-1 interaction with one C1q collagen stem (Figure 8B and Figure S3). Based on our results and the constraint of a small enough residue in front of the LAIR-1 I102, the most relevant model is the one represented in Figure 8B. This model unveils two C1q residues potentially engaged in LAIR-1 docking: arginine 72 and lysine 65 on C1q A and B chains, respectively. These two CLR residues were mutated with a view to confirm their involvement in LAIR-1 binding. However, the generated C1qCLR_nc2 fusion protein variants exhibited no or slight inhibitory effect on LAIR-1 interaction, which could be expected. Indeed, as mentioned above, the A_R72 and B_K65 CLR residues, in association with the respective E111 and E72 LAIR-1 residues, are not involved in generic collagen backbone recognition of C1q by LAIR-1 but are suggested to play a role in the recognition specificity and the correct positioning of both partners. Interestingly, the collagen sequence of C1qC chain recognized in our model is exposed on the same side as the two most important lysine residues of C1q for the interaction with the C1r_2_s_2_ tetramer (Figure 8B). This suggests that the LAIR-1 Ig-like domain may interact with one C1q collagen stem close to and with the same orientation as C1r_2_s_2_, therefore explaining the strong competition effects observed in Figure 2B.

However, we cannot exclude that LAIR-1 might interact with C1q through the two other putative binding sites (Figure S3). These two sites are located on the C1q B chain, far enough from the motif in the C chain to accommodate the binding of one LAIR-1 Ig-like domain on each site at the same time. Such simultaneous binding of two LAIR-1 copies on C1q collagen stem could be consistent with the required dimerization of LAIR-1 to trigger its inhibitory activity. Indeed, the dimerization of LAIR-1 upon ligand binding results in the phosphorylation of its ITIMs motifs, leading to a negative regulation of intracellular signaling associated with immune cell maturation, activation and differentiation [10,37,38,39]. Therefore, the dimerization of LAIR-1 might be mediated by the binding of two copies on either the same site on two different C1q collagen stems or two different sites on the same C1q collagen stem.

## 4. Materials and Methods

### 4.1. Proteins, Cells and Reagents

C1q was purified from human serum as described previously [40]. C1q collagen-like and globular regions were prepared as described in Tacnet-Delorme et al. [41]. Recombinant C1r_2_s_2_ tetramer (C1s, C1rS637A) was purified and quantified according to published procedures [42]. Recombinant C1q variants were produced and purified as reported in Bally et al. [20].

Oligonucleotides were purchased from Eurogentec (Liege, Belgium), restriction and modification enzymes from New England Biolabs.

### 4.2. Construction of the C1qCLR_nc2 Protein

Recombinant C1qCLR_nc2 (WT and mutants) was produced and purified as previously reported [21] with the difference that L-ascorbic acid 2-phosphate was added during the production step in the EXPI culture medium at a final concentration of 450 µM and the concentration of L-ascorbic acid was reduced to 250 µM (instead of 568 µM in Fouët et al. 2020a). Previous reports showed that L-ascorbic acid 2-phosphate is more stable than L-ascorbic acid and enhances production yields for some collagen types [43]. The DNA sequences corresponding to the GPP triplets present between the C1qCLR and nc2 sequences were removed by site-directed mutagenesis with the Quickchange II XL kit (Agilent technologies (Les Ullis, France).

### 4.3. Construction and Purification of the C1qstem_nc2 Protein

The coding sequences of C1q native signal peptides followed by CLRA (40–87, mature numbering) fused to nc2α2, CLRB (42–89, mature numbering) fused to nc2α1, and CLRC (39–86, mature numbering) fused to nc2α3 with a C-terminal FLAG epitope were synthesized and cloned in pcDNA3.1/Neo(+), pcDNA3.1/Hygro(+), and pcDNA3.1/Zeo(+) vectors, respectively (Thermo Fisher Scientific, Illkirch, France). The C1qstem_nc2 fragment (WT and mutants) was produced in expi293 cells (Thermo Fisher Scientific) and first purified on an anti-FLAG M2 affinity column (Sigma-Aldrich, St Quentin Fallavier, France) as described for the full length CLR_nc2 [21]. The second purification step was performed on a Superdex 75 10/300 column (GE Healthcare, Velizy-Villacoublay, France).

### 4.4. LAIR-1 Ig-Like Domain Expression and Purification

The C2-type Ig-like domain sequence of LAIR-1 from residues 22 to 122 was inserted in a pET28a plasmid for bacterial expression with a polyhistidine tag at the C-terminal extremity to allow protein purification. Soluble LAIR-1 Ig-like domain was overexpressed by pET28a-LAIR-1 transformed *E. coli* BL21(DE3) cells using an autoinduction protocol [44]. LAIR-1 Ig-like domain was purified in a two-step process. First, it was affinity purified on a nickel-loaded hitrap chelating HP column (GE Healthcare), washed by a 35 mM imidazole step followed a 35–500 mM imidazole gradient in 25 mM Tris, 150 mM NaCl (pH 8.2). Then a gel filtration step was performed using a Superdex 75 column (GE Healthcare) in 25 mM Tris, 150 mM NaCl (pH 8.2). The LAIR-1 mutants were expressed and purified with the same experimental procedures than for the wild-type. At each purification step, LAIR-1 concentration was estimated using an A_1%,1cm_ of 10.3 and the molecular masses presented in Figure 1A.

### 4.5. SDS/PAGE, Circular Dichroism and LC/ESI Mass Spectrometry

#### 4.5.1. SDS/PAGE

Recombinant LAIR-1 was analyzed by SDS/PAGE under reducing conditions using 14% poly-acrylamide Tris/HCl gels and then colored using Instant-Blue (Expedeon, Abcam, Paris, France).

#### 4.5.2. Circular Dichroism

The far-UV circular dichroism (CD) spectra (from 204 to 260 nm) was collected on a JACSO J-810 CD spectrophotometer. LAIR-1 variants samples at a concentration of 4 µM in 5 mM Trizma, 150 mM NaF (pH 8.2) were scanned 10 times at 20 °C in a 1 mm path-length cuvette. After subtraction of baseline buffer values, the CD signal was converted to mean molar residue ellipticity (deg.cm^2^.dmol^−1^).

#### 4.5.3. LC/ESI Mass Spectrometry

Liquid Chromatography Electrospray Ionization Mass Spectrometry (LC/ESI-MS) analyses were performed on a 6210 LC-TOF spectrometer coupled to a HPLC system (Agilent Technologies).

All solvents used were HPLC grade (Chromasolv, Sigma-Aldrich), trifluoroacetic acid (TFA) was from Acros Organics. Solvent A was 0.03% TFA in water, solvent B was 95% acetonitrile, 5% water, 0.03% TFA. Before analysis, protein samples were diluted in acidic denaturing conditions to a final concentration of 10 µM with solvent A. Protein samples were firstly desalted on a reverse phase-C8 cartridge (Zorbax 300SB-C8, 5 mm, 300 Å 5*0.3 mm, Agilent Technologies) and then eluted with 95% solvent B.

MS spectra were recorded in the positive ion mode in the 300–3200 m/z range and the data processed with MassHunter workstation software (v. B.02.00, Agilent Technologies).

### 4.6. Surface Plasmon Resonance Analyses and Evaluation

All SPR experiments were done at 25 °C on a BIAcore T200 instrument (GE Healthcare). Each ligand was immobilized on a CM5 sensor chip using the amine coupling chemistry at a flowrate of 10 µL/min in 10 mM HEPES, 150 mM NaCl, 3 mM EDTA, 0.05% (v/v) surfactant P20 (pH 7.4) according to the manufacturer’s protocol (GE Healthcare). sC1q or LAIR-1 were diluted at 35 µg/mL in 10 mM sodium acetate (pH 5) or 5 µg/mL in 10 mM sodium acetate (pH 4.5), respectively, for the immobilization procedure. All other ligands were immobilized at a concentration of 25 µg/mL in 10 mM sodium acetate (pH 5). All SPR interaction experiments were performed at a flowrate of 30 µL/min in 50 mM Tris, 150 mM NaCl, 2 mM CaCl_2_, 0.05% surfactant P20 (pH 7.5). All data were analyzed using the T200 Biaevaluation 3.1 software (GE Healthcare).

Since the aim of our project was to compare several different LAIR-1 mutants for their interaction with C1q variants, we decided to choose the orientation that allowed precise comparison and reproducibility. We therefore chose the orientation in which C1q and variants are immobilized and all the interactions with LAIR-1 mutants are analyzed in the same conditions and on the same C1q surfaces.

### 4.7. LAIR-1 Interaction with sC1q, sCLR and sGR and Competition Assay with C1r_2_s_2_

LAIR-1 Ig-like domain was injected at a concentration of 10 µM on immobilized sC1q (14,000 RU), sCLR (6000 RU) or sGR (1500 RU) with an association step of 180 s.

For the competition assay, sC1q (1 nM) was preincubated with C1r_2_s_2_ at different molar ratios in 50 mM Tris, 150 mM NaCl, 2 mM CaCl_2_, 0.05% surfactant P20 (pH 7.5) (15 min at 25 °C) before injection over immobilized LAIR-1 Ig-like domain (330 RU) with an association step of 180 s.

### 4.8. Interaction of LAIR-1 Variants with Immobilized Serum-Derived C1q

Wild-type and mutated LAIR-1 Ig-like domains were injected at a concentration of 10 µM over immobilized sC1q (14,000 RU) with an association step of 180 s. The responses obtained at the equilibrium for each LAIR-1 mutant were normalized as a percentage of the WT interaction signal.

### 4.9. Kinetics of LAIR-1 Interaction with sC1q, rC1q Variants and CLR_nc2 Variants

Soluble LAIR-1 Ig-like domain was injected at increasing concentrations (4–128 µM) over immobilized sC1q (14,000 RU), rC1q WT (12,000 RU) and rC1q ABC (12,000 RU) with an association step of 180 s. The equilibrium dissociation constant (*K*_D_) values were calculated from measured responses at equilibrium (Req) by fitting plots Req versus concentration using steady state analysis (Biaevaluation 3.1 software).

Soluble LAIR-1 was injected at increasing concentrations (4–256 µM) over immobilized C1qCLR_nc2 WT (6800 RU), C1qCLR_nc2 A_R72E (6800 RU) and C1qCLR_nc2 B_K65E (6400 RU) with an association step of 60 s. LAIR-1 was injected at different concentrations (3.125–400 µM) over immobilized C1qstem_nc2 (2600 RU) with an association step of 30 s.

The equilibrium dissociation constant (*K*_D_) values were calculated from measured responses at equilibrium (Req) by fitting plots of Req versus concentration using steady state analysis (Biaevaluation 3.1 software).

### 4.10. NanoDSF Experiments

NanoDSF experiments were performed using a Prometheus NT.48 instrument (Nanotemper Technologies) and the provided software PR.thermocontrol v2.0.4. Up to 48 capillaries containing 10 μL of sample were sequentially illuminated at 280 nm, and fluorescence intensity at 350 (F350) and 330 (F330) nm was measured at increasing temperatures (20–95 °C, 1 °C/min). The F350/F330 ratio and its first derivative were then plotted versus temperature.

NanoDSF analyses were performed on wild-type and mutated LAIR-1 Ig-like domain at a concentration of 80 µM alone or in presence of 80 µM of a synthetic triple helical collagen peptide in order to detect the collagen peptide binding to LAIR-1. The synthetic collagen peptide with the sequence Ac-GPOGPOGPOGPKGEQGPOGPO-NH2 was purchased from GL Biochem (Shanghai, China).

## Figures and Tables

**Table 1:** Equilibrium dissociation constants for the binding of LAIR-1 WT to C1q and C1q variants.

Immobilized Ligands	LAIR-1 WT *K*_D_ (µM)
sC1q	112.3 ± 6.2 (n = 4)
rC1q WT	99.5 ± 26.6 (n = 2)
rC1q ABC	131.8 ± 23.5 (n = 2)
C1qCLR_nc2	179.3 ± 4.8 (n = 3)
C1qCLR_nc2 AR72E	295.0 ± 10.6 (n = 3)
C1qCLR_nc2 BK65E	188.8 ± 5.3 (n = 3)
C1qstem_nc2	174.9 ± 0.6 (n = 2)

Values are means ± SD from separate experiments. The number of replicates (n) are indicated next to the *K*D values. The dissociation constant *K*D was determined by global fitting of the data using a steady-state binding model.

## Supplementary Materials

The following are available online at https://www.mdpi.com/article/10.3390/ijms22105125/s1, Figure S1: Comparison of bacterial and mammalian recombinant LAIR-1 extracellular region, Figure S2: Far-UV circular dichroism spectra (from 204 to 260 nm) of LAIR-1 WT (in blue), R59A (in green) and E61A (in red), Figure S3: Alternative models of LAIR-1 Ig-like domain in complex with C1q collagen stem, Figure S4: Supplementary GPP triplets introduce additional LAIR-1 binding sites within CLR_nc2, Figure S5: Binding assay of a control collagen peptide on LAIR-1 mutants by NanoDSF experiments.
