# Supplementary material for: Molecular Basis of Complement C1q Collagen-Like Region Interaction with the Immunoglobulin-Like Receptor LAIR-1
Source: Int J Mol Sci. 2021 May 12;22(10):5125. doi: 10.3390/ijms22105125 (PMC8151509; doi:10.3390/ijms22105125)

Figure S1. Interaction of bacterial and mammalian recombinant LAIR-1 extracellular regions with C1q. LAIR-1 produced in *E. coli* BL21 (red curve) or in HEK293F cells (green curve) was injected at a concentration of 8 µM over immobilized serum C1q (14,000 RU).

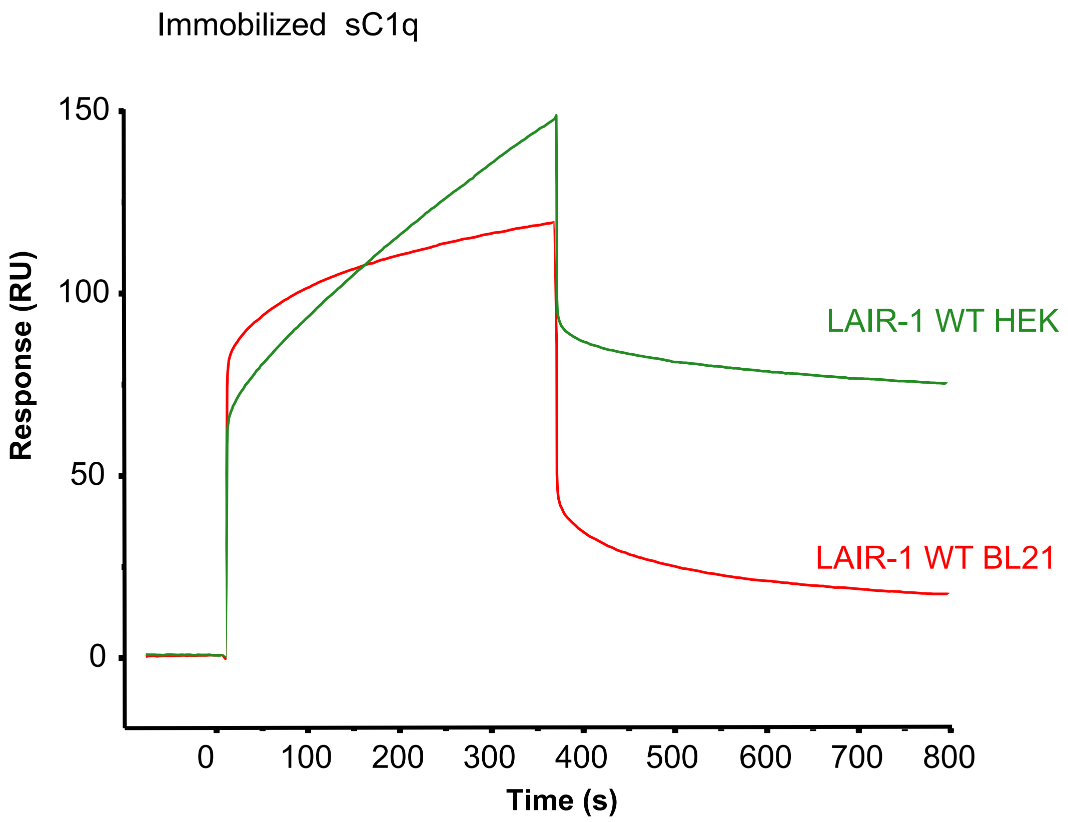

Figure S2. Far-UV circular dichroism spectra (from 204 to 260 nm) of LAIR-1 WT (in blue), R59A (in green) and E61A (in red).

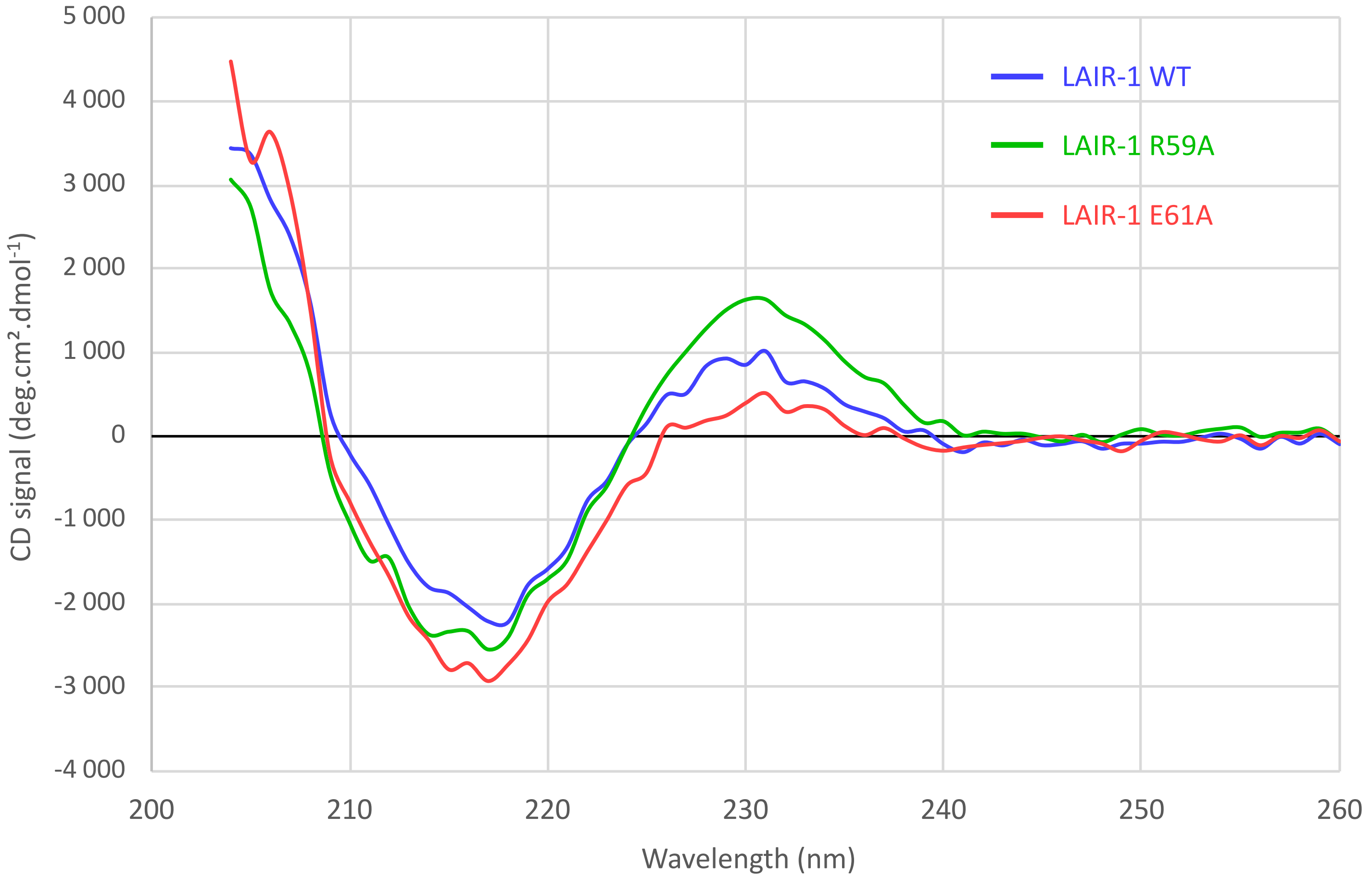

Figure S3. Alternative models of LAIR-1 Ig-like domain in complex with C1q collagen stem. Models of LAIR-1 Ig-like domain (blue, PDB code: 3KGR) in interaction with the C1q CLR triple helix (A chain in cyan, B chain in pink and C chain in green). The collagen-binding groove of LAIR-1 is shown in grey and the side chains of the identified key binding residues are shown and labelled. Remarkable lysine 65 and arginine 72 of C1q B and A chains respectively are labelled. The residues facing the I102 of LAIR-1 in each model are labelled in black. The side chains of the identified OGXO motifs are shown in lines.

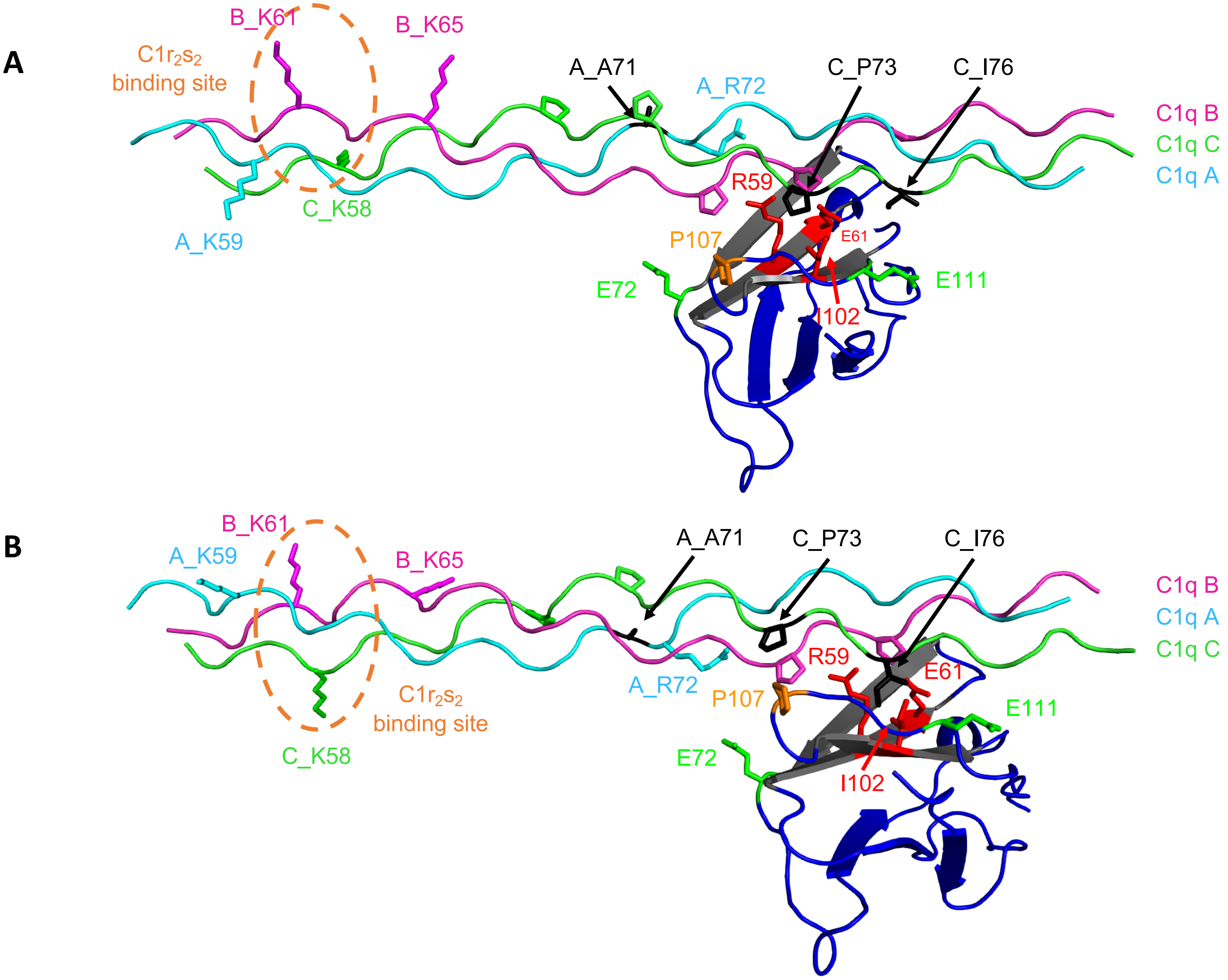

Figure S4. Supplementary GPP triplets introduce additional LAIR-1 binding sites within CLR_nc2. (A) LAIR-1 was injected at increasing concentrations (4-128 µM, 2-fold serial dilution) over immobilized CLR_nc2 containing the GPP triplets between the CLR and nc2 sequences. Fits obtained by a global fitting of the data to a steady-state model are shown in the top right corner. The result shown is representative of separate experiments (see B). (B) Summary table of the Rmax and K_D values derived from the SPR kinetic analyses obtained with the old (with GPP) and new (without GPP) generation of the CLR_nc2 fusion protein.

A

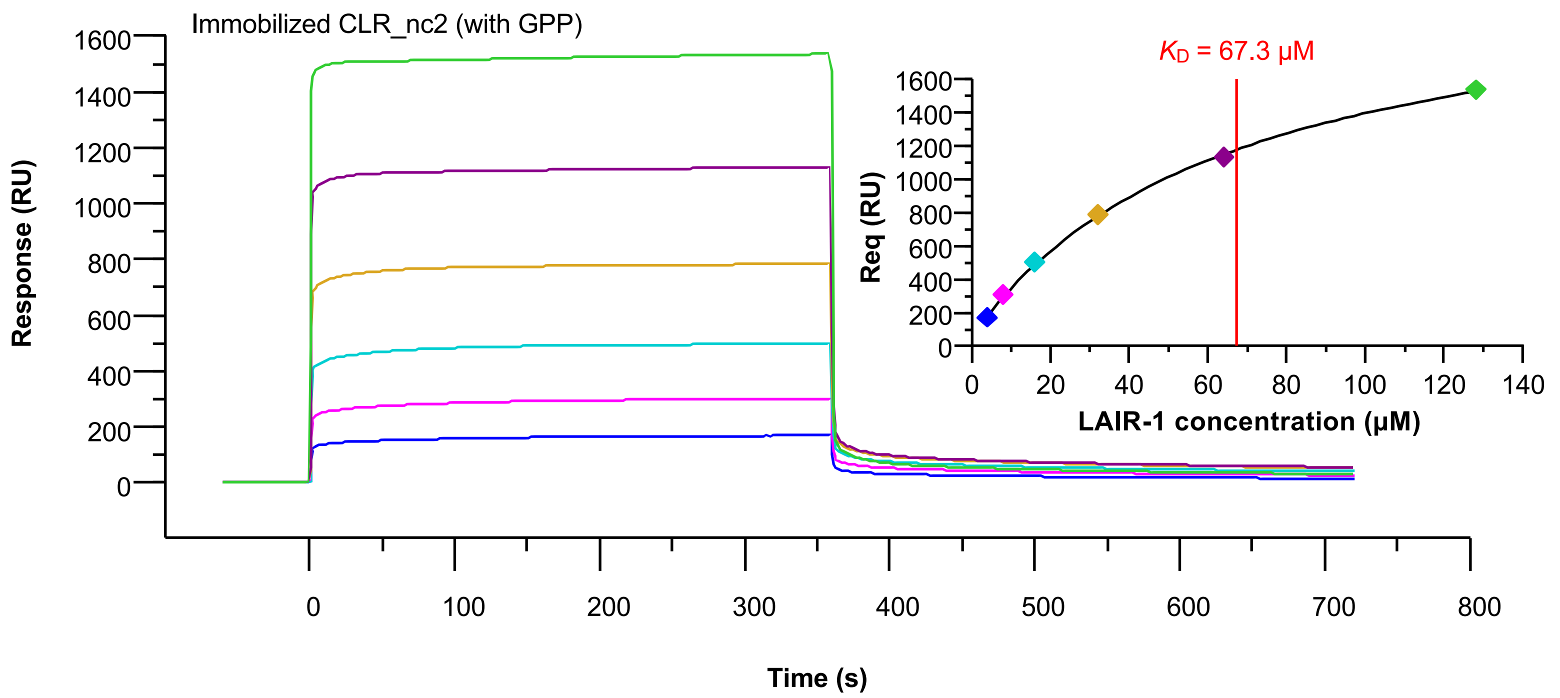

B

Ligand	LAIR-1	
	Rmax (RU)	K_D (µM)
sC1q	1443 ± 31	112.3 ± 6.2 *(n=4)*
CLR_nc2 (with GPP)	2325 ± 168	79.5 ± 11.6 *(n=3)*
C1qCLR_nc2 (without GPP)	871 ± 18	179.3 ± 4.8 *(n=3)*

Values are means ± SD from separate experiments. The number of replicates (n) are indicated next to the K_D values
The dissociation constant K_D was determined by global fitting of the data using a steady-state binding model.

Figure S5. Binding determination of a control collagen peptide on LAIR-1 mutants by NanoDSF experiments. Analysis of LAIR-1 WT (in green), I102L (in orange), I102Y (in blue), E72K (in red), E111K (in black) and P107R (in purple), alone (dashed curves) or in the presence of a control collagen peptide (solid curves). The horizontal dashed lines indicate the ranges considered for the results in Figure 7B. The horizontal green dashed line delimit the signal that was considered as an interaction (+) and the red dashed line indicates the limit of what was considered as no interaction (-). In between these two lines the interaction of the collagen peptide was considered as intermediary (±). The same limits were also used for the mutants of Figure 5. The red star indicates the signal for the control collagen peptide interaction.

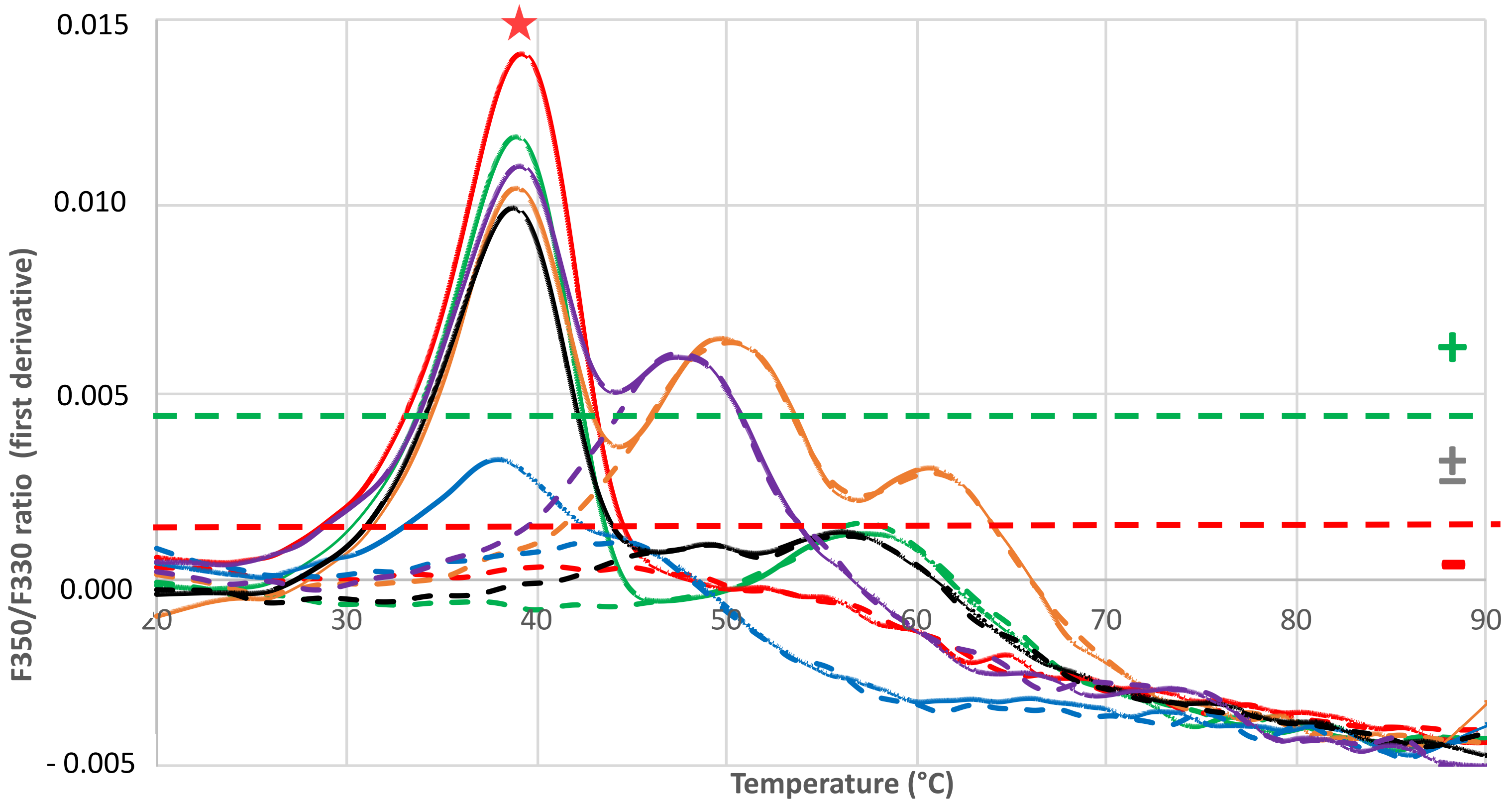

Supplement: Supplementary file 1 [file ijms-22-05125-s001.zip › ijms-1138221-supplementary.pdf]
